# The relationship between home- and individual-level diet quality among African American and Hispanic/Latino households with young children

**DOI:** 10.1186/s12966-018-0645-9

**Published:** 2018-01-15

**Authors:** Angela Kong, Linda Schiffer, Mirjana Antonic, Carol Braunschweig, Angela Odoms-Young, Marian Fitzgibbon

**Affiliations:** 1 Department of Kinesiology and Nutrition, University of Illinois at Chicago, 1919 W Taylor St, Chicago, IL 60612 USA; 2 Institute for Health Research and Policy, University of Illinois at Chicago, 1747 W Roosevelt Rd, Chicago, IL 60608 USA; 3 Department of Pediatrics, University of Illinois at Chicago, 1835 W Polk St. Chicago, Chicago, IL 60612 USA; 4 University of Illinois Cancer Center, 914 S. Wood St. MC 700, Chicago, IL 601612 USA

**Keywords:** Diet, Healthy eating index, Households, Children, African American, Hispanic

## Abstract

**Background:**

The quality of most Americans’ diets is far from optimal. Given that many Americans consume a significant portion of calories in the home, intervening in this setting could be beneficial. However, the relationship between the home food environment and diet quality is not well understood. This study examined the relationship between diet quality at the individual level with home-level diet quality using an index that measures compliance with federal dietary guidance.

**Methods:**

This was a cross sectional study that enrolled 97 African American and Hispanic/Latino low-income parent-child dyads. Diet quality at the individual level was assessed through two 24-h dietary recalls collected for parents and children, respectively. Diet quality at the home level was assessed with two home food inventories conducted in participants’ homes. Diet quality scores at the home and individual levels were computed by applying the Healthy Eating Index-2010 (HEI-2010) to these data. Linear models adjusted for potential confounding factors were used to examine the relationship between diet quality at the home and individual levels.

**Results:**

Total HEI-2010 scores from parents and children’s diets were positively associated with HEI-2010 scores based on home food inventories (parent diet: β: 0.36, 95% CI: 012–0.60; child diet: 0.38 95% CI: 013–0.62). Positive associations were also observed between individual level and home level subcomponent HEI-2010 scores for total fruit (parent: 0.55 95% CI: 0.16–0.94; child: 0.49 95% CI: 0.03–0.94), whole fruit (parent only: 0.41 95% CI: 0.07–0.74), greens and beans (parent only: 0.39 95% CI: 0.05–0.74), and whole grain (children only: 0.33 95% CI: 0.04–0.63).

**Conclusion:**

This study demonstrated that individual level diet quality was positively associated with home-level diet quality. Findings from this study can help us to address modifiable targets of intervention in the home to improve diet quality.

## Background

Findings from many large prospective cohort studies offer consistence evidence that higher diet quality, as measured by diet quality indices such as the Healthy Eating Index(HEI), are associated with lower all-cause and cause specific mortality [1–4] and less risk of cardiovascular disease [2, 3] and various cancers [2, 5–11]. However, nationally representative data reveals that most US children and adults do not adhere to dietary patterns promoted by the Dietary Guidelines for Americans [12, 13] and further disparities in diet quality are observed among some subgroups [14–16]. For instance, diet quality scores, as measured by the Healthy Eating Index-2010 (HEI-2010), were consistently lower among Non-Hispanic Black children compared to other racial/ethnic groups across many cycles (1999–2012) of data from the National Health and Nutrition Examination Survey (NHANES) [14]. Additionally, low-income individuals (≤ 130% of the poverty threshold) were less likely to meet recommendations for key HEI components such as fruits, vegetables, and whole grains when compared to higher income individuals (>185% of the poverty threshold) [15].

Many determinants (e.g. social, environmental, economic, political, etc.) contribute to the disparities observed in diet quality [17, 18]. Therefore, a greater understanding of contextual factors may advance understanding of this problem. Increasingly, evidence supports the home food environment as playing a role in the diets of families, especially for those with preschool-age children [19–21]. Based on NHANES data, ‘foods consumed at home’ is a significant contributor of calories among households with children in this age group [22]. In particular, home food availability (i.e. availability of select foods in the home) has been identified as an important determinant of dietary intake [23–26] . To date, most studies that assess home food availability (HFA) have largely relied on checklists (both self-reported and observed) [27–31]. Checklists measure availability of healthful foods (e.g. fruits and vegetables) [24, 27, 32] and unhealthful foods (e.g. sweets, chips, sugar sweetened beverages) [28, 29, 33, 34]. However, checklists may miss foods regularly available in homes of less studied groups (e.g., racially/ethnically diverse groups, families with preschool-aged children, etc.) because they are limited by a pre-defined food list. Furthermore, overall diet quality cannot be easily assessed using this approach. While labor and time intensive, exhaustive home food inventories are made more feasible through barcode scanning [35–37] and can address some of the limitations found with checklists. For instance, this method does not rely on self-report or require researchers to make assumptions about the home availability of foods since it assesses what foods are actually in the home rather than how well a household conforms to a checklist [35, 36]. This approach, in its comprehensiveness (i.e. all foods) and precision (i.e. specific foods, brands, amounts) offers a flexible approach for capturing any nutrition-related dimension of interest, including diet quality.

Of the studies that have tested this type of approach [35–41], none has directly examined the diet quality of the home food supply in relation to individual-level diet quality. Establishing this link is a vital step in identifying modifiable targets for home based intervention. Unlike more distal food environments (e.g. corner stores, grocery stores, national food supply), the home food environment is a target that can be immediately addressed in a feasible manner by households. Furthermore, there are undoubtedly variations across households and having a way to measure differences can allow for more targeted approaches to intervention. Therefore, the purpose of this study was to examine the relationship between individual-level total diet quality (based on the diets of parents and children) and home-level total diet quality (based on home food inventories) among low-income minority families with preschool-age children. As a secondary outcome, we also examined subcomponents of diet quality at the individual and home levels.

## Methods

### Participants

Parent-child dyads living in Chicago, Illinois were recruited from November 2014 through March 2016. Participants were enrolled in The Study on Children’s Home Food Availability using TechNology (SCAN), a cross sectional study examining the home food environments of low-income African American and Hispanic/Latino households with preschool-age children. A purposive sample was recruited through brochures and flyers advertised in clinics and/or schools offering Head Start or the Special Supplemental Nutrition Program for Women, Infants, Children (WIC) located throughout the city of Chicago. These sites were chosen specifically because they were most likely to reach this study’s target population of interest (i.e. low-income African American and Hispanic/Latino parents with children from 2 to 5 years). Interested parents contacted study staff and were screened for eligibility over the phone. Eligibility criteria considered both the parent and child since parent-child dyads were enrolled in the study. Parent-child dyads were excluded if: 1) the child was not between 2 and 5 years of age, 2) parents or caregivers did not self-identify as African American/Black or Hispanic/Latino, 3) parent was not fluent in English or Spanish 4) child (2–5 years) did not live with the parent or caregiver regularly (at least 4 days a week) 5) the child had a disability or illness that significantly altered dietary intake and required a medically prescribed diet, 6) parent-child dyad would not be available during the next month, 7) parent was pregnant past the first trimester.

**Fig. 1:**
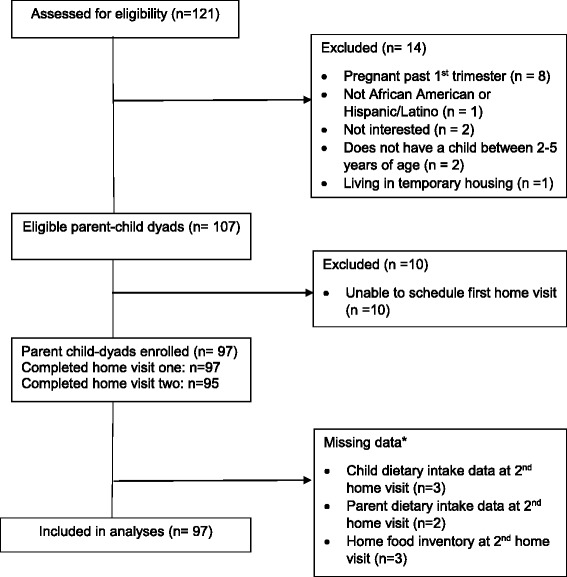
Participant flow diagram. *Values from 1st home visit used in analyses instead of mean of visits 1 and 2

A total of 121 parent-child dyads were screened for eligibility and of those, 107 were eligible (88%) (Fig. 1). Of those meeting eligibility criteria, 97 parent-child dyads (91%) enrolled in the study and written informed consent was obtained for all participants at the first home visit. This exceeded our expected sample size of 80 parent-child dyads (i.e. sample size of 90 with 90% retention). We determined that a sample size of 80 parent-child dyads (40 dyads in each racial/ethnic group) would have sufficient power (β = 0.80, α = 0.05) to detect a medium effect size [42] of *r* = 0.52 with adjustment of five covariates within the model. All parent-child dyads were included in the analytic sample; however, some data were missing for a limited number of households at the second home visit only (Fig. 1). For instance, home food inventories were not completed in three households and diet recalls were missing for three children and two parents, respectively. In these households, available data from home visit one was used in estimates instead of averaging data from two home visits. Study procedures were approved by the University of Illinois at Chicago Institutional Review Board.

### Data collection procedures

#### Home visits

Trained research staff collected all of the data. Hispanic/Latino participants were interviewed by bilingual interviewers (fluent in English and Spanish) in the language of their choice. Data were collected during two home visits scheduled approximately two weeks apart. Some of the measures collected at the first home visit included anthropometrics, sociodemographic information, eating-related habits and practices, dietary intake (parent and child), and home food inventories. The second home visit again assessed dietary intake (parent and child), and home food inventories. Participants received $65 for the first home visit and $45 for the second home visit.

#### Sociodemographic variables

Trained research staff interviewed participants to collect sociodemographic information such as race/ethnicity, age, education level, household income, marital status, employment status, household size, and use of food assistance programs.

#### Acculturation

To assess acculturation status, a 4-item brief acculturation scale was administered to parents who identified as Hispanic/Latino. This scale is based on the *language use* factor of the Short Acculturation Scale for Hispanics developed by Marin and colleagues [43]. This measure demonstrates good reliability (α =0.90) and is moderately correlated with generation (*r* = 0.67) and length of time in the US (*r* = 0.50), which are used to assess construct validity for this measure [44]. These estimates are comparable to those found with the original measure [43, 44]. This scale ranges from 1 (least acculturated) to 5 (most acculturated) and is based on an average of the four items. Scores of greater than 3 indicate higher levels of acculturation.

#### Food security

The USDA’s Six Item Short Form of the US Food Security Module was used to assess food security [45]. The six-item scale is an efficient way to evaluate food security and classifies food security status with reasonable sensitivity (88.5%) and specificity (99.5%) among households with children [46]. Respondents are asked to answer questions based on the food eaten in their household in the last 12 months and whether or not they could afford the food they needed. Scores are classified in one of three categories: high or marginal food security (score: 0–1), low food security (score: 2–4), or very low food security (score: 5–6). Food security scores are based on the number of affirmative responses.

#### Family eating habits and food shopping habits

As part of the interview, data collectors also obtained information on family eating habits and food shopping habits. Based on a question from Project F-EAT [47–49], we asked parents: “During the *past week,* how many times was a family meal purchased from a fast food restaurant and eaten together either at the restaurant or at home (pizza counts)?” Response categories included “never”, “1 time”, “2 times”, “3 or more times”. A question about shopping habits asked: “Thinking about the past month, how often do you food shop for the family?” Response categories included “less than once a month”, “once a month”, “twice a month”, “three times a month”, “four times a month or more”.

### Anthropometrics

Height and weight measurements for both parents and the children were obtained by trained staff at the first home visit. Weight was measured on a SECA 214 portable digital weight scale (SECA, Hanover, MD), and height was measured using a BWB-800 portable stadiometer (Tanita Corp., Arlington Heights, IL). Both height and weight were measured twice and then averaged together. A 0.5 cm difference between the two height measurements or a 0.2 kg difference in weight required a third measure to be taken and the mean of the two closest measurements were used. BMI was then calculated as weight (kg)/[height(m)]^2^.

### Dietary intake

Diets of parent-child dyads were assessed at each home visit through 24-h diet recalls (one recall per person). Twenty-four hour recalls based on parent report has been previously validated in this age group (i.e. preschool) [50–52]. If the 24 h recall period occurred on a day that the child was at childcare, parents were asked to gather information from the childcare provider regarding the foods offered and consumed. Parents notified childcare providers ahead of time to make notes on the menu regarding the foods the child selected and consumed.

A master’s level research dietitian, previously certified by the Nutrition Coordinating Center at the University of Minnesota to conduct 24-h diet recalls, trained the data collection staff and monitored recalls for quality. Data collectors conducted recalls through a guided, automated, multi-pass approach using Nutrition Data for Research (NDSR) software version 2014 (University of Minnesota). Standardized amount booklets assisted in estimating portions and Spanish-translated versions of the materials (e.g. interviewer prompts and amount booklets) were used when appropriate.

### Home food inventories

Home food inventories were conducted at both home visits approximately two weeks apart in an attempt to capture the intra-household variability of the home food supply in a typical month. Previous research using a similar approach (i.e. home food inventories via barcode scanning) suggests that two inventories within the course of a month may be adequate to capture the usual macronutrient characteristics of a household [53]. Home food inventories were scheduled on all days of the week; however, appointments were not scheduled within one week of major holidays to better reflect the usual household food supply. Home food inventories were also conducted across all seasons to minimize the influence of seasonal variation on the home food supply. Both foods with and without universal product codes (UPC) were inventoried, including leftovers and other home-prepared foods. The only items that were not inventoried were pet foods, infant formulas and breast milk, commercially prepared infant foods or pre-made baby foods, herbs/spices, non-caloric condiments, tea bags/leaves, coffee beans, individual packets of condiments, alcoholic beverages, cooking sprays, dietary supplements, sugar free gum or mints, cough drops, and foods used for decoration (e.g. Jack O Lantern).

#### Inventory of barcode items

The data collection staff received extensive training, led by the principal investigator (PI), on barcode scanning procedures. Prior to data collection, the study staff practiced scanning food items (~50 food items) in a controlled setting to gain familiarity with the process. Foods scanned by staff members were compared to foods scanned by the PI and training continued until 100% concordance was achieved. Data collectors also trained in two practice homes in order to simulate real world conditions. Each data collector was assigned to a food storage area and the PI observed each person to ensure scanning procedures were followed according to study protocol.

A commercially available app (Prep and Pantry, version 3.7.01, © 2011–2016 Mark Patrick Media LLC; last updated July 18, 2016) installed on an iPad mini 2 (Apple Inc., Cupertino, CA) was used to inventory all foods with UPCs. Data collectors scanned these food items into the app and entered the following data for each food item: 1) food item/brand name, 2) net weight, 3) quantity, 4) calories per serving. Data collectors also photographed the front and back of each food item to further verify food items collected by the app. Digital photographs captured 1) food item and brand name, 2) net weight, 3) nutrition facts panel information, and 4) universal product codes. All inventoried barcode items were organized in table format and exported as a CSV (comma-separated values) file that was backed up after each visit. All barcode items were entered into NDSR software (version 2014) to be later analyzed for nutrient and diet quality. Data from the CSV file and corresponding photos aided research staff in identifying the exact foods item and/or comparable matches in NDSR.

#### Inventory of non-barcode items

Non-barcode items were assessed separately on a log form that described the food item and the amount (net weight and quantity). When data collectors encountered unfamiliar foods, they would gather additional information from participants regarding the ingredients and preparation of the food item. A digital photograph with a label of the food item also accompanied each non-barcode food item on the log form. A log was kept for each household at each visit, respectively. Some examples of non-barcode items included produce, bulk items (e.g. nuts and seeds), deli items, home-prepared foods, and ready to eat foods purchased from restaurants or grocery stores. Similar to barcode items, non-barcode items were also entered into NDSR for nutrient analyses. Log entry descriptions and corresponding digital photographs assisted in the data entry of these food items in NDSR.

#### Diet quality of individual dietary intake and home food supply

All food items (barcode and non-barcode) from home food inventories and diet recalls were entered in NDSR for nutrient and food group analyses and assessment of diet quality. NDSR is a widely-used nutrient database in nutrition research consisting of more than 18,000-food items including 8000 brand names (University of Minnesota). The USDA National Nutrient Database for Standard Reference [54] and the Food and Nutrient Database for Dietary Studies (FNDDS) [55] serve as underlying databases for NDSR and updates from these databases are incorporated into NDSR on an ongoing basis [56]. Furthermore, the food coding system in NDSR is modeled after the USDA food coding scheme and the food group serving count system is modeled after USDA Food Patterns Equivalents [57].

Diet quality was assessed using the Healthy Eating Index 2010 (HEI-2010). HEI-2010 measures diet quality based on compliance with the 2010 Dietary Guidelines for Americans and demonstrates adequate construct and concurrent criterion related validity and reliability [58]. This diet quality index consists of twelve components: total fruit, whole fruit, total vegetables, greens and beans, whole grains, dairy, total protein foods, seafood and plant proteins, fatty acids, refined grains, sodium, and empty calories. HEI-2010 uses a density approach which measures diet quality independent of energy intake which makes it an appropriate measure to evaluate diet quality at the individual-level [58, 59] and at the level of the environment [60–63]. Except for empty calories and fatty acids, HEI-2010 components are calculated based on a standard unit (e.g. cup or ounce equivalents) per 1000 kcal (kcals). Empty calories are expressed as a percentage of total calories and fatty acids are expressed as a ratio of polyunsaturated fats (PUFA) and monounsaturated fats (MUFA) to saturated fats (SFA)(PUFA + MUFA/SFA). The total HEI score is the sum of all component scores with a maximum value of 100. Higher scores reflect better compliance with the dietary guidelines.

To assess diet quality, HEI-2010 scoring standards were applied to 24-h diet recall data and home food inventory data using the same approach. To compute total HEI and subcomponent scores, foods were disaggregated into component ingredients; these ingredients were then assigned to corresponding food groups (e.g. fruits, vegetables, added sugars, solid fats meats, grain, etc.); and food groups were converted into food pattern equivalents so that units reported conformed with the HEI (e.g. ounce equivalents, cup equivalents, etc.).

HEI-2010 total and subcomponent scores were estimated from the average of two 24-h diet recalls based on parents and children’s diets, respectively. To assess the diet quality of the home food supply, HEI-2010 total and subcomponent scores were estimated based on the average of two home food inventories conducted in each household. All inventoried items were included in the calculations. Previous research suggests that estimates are not unduly influenced by items such as drink mixes, pantry staples (e.g. oil, flour, sugar, etc.), and other bulk items [62]; therefore, those items remained.

### Statistical analyses

Descriptive statistics, presented as means or proportions as appropriate, were calculated to describe the study sample and to summarize diet quality indices. Summary statistics of HEI-2010 total and subcomponent scores by race/ethnicity, including % maximum score ([mean score/ maximum score] × 100%), were reported for parents, children, and the home food supply. Linear models were used to examine the relationship between individual-level diet quality (dependent variable) and the diet quality of the home food supply (independent variable). The main dependent and independent variables were treated as continuous variables. Residuals were checked for normality (based on quantile-quantile plot) along with other model assumptions. The potential moderating effect of race/ethnicity was tested by including an appropriate interaction term; however, this term was not significant. Models controlled for race/ethnicity, age, education, marital status, employment status, income, household size, participation in food assistance programs, household food security status, grocery shopping frequency, fast food meal frequency, and body mass index (BMI). All analyses were conducted using STATA version 13.1 (StataCorp LLC, College Station, TX).

## Results

### Sociodemographics

Table 1 provides a description of the study sample. There were 97 Hispanic/Latino and African American parent-child dyads (Hispanic/Latino *n* = 47, African American *n* = 50) enrolled in this study. Parents were on average 33.4 years (SD: 8.0), primarily mothers (97.9%), and 47.4% of the sample had a high school degree or less. A greater proportion of Hispanic/Latina mothers reported being married or living with a partner (74.5%) compared to African American parents (18.0%). The majority of children in the sample were boys (63.9%) with a mean age of 47.1 months (SD: 15.5).

**Table 1:** Sociodemographic and Anthropometric Characteristics of SCAN Study Participants by Race/Ethnicity

	Total (*n* = 97)	African American (*n* = 50)	Hispanic (*n* = 47)
Parent Age(yrs) *mean (sd)*	33.4 (8.0)	32.4 (8.6)	34.4 (7.3)
Child Age (mos) *mean (sd)*	47.1(15.5)	50.2(15.4)	43.8 (15.1)
Female % (n)			
Parent	97.9% (95)	98.0%(49)	97.9%(46)
Child	37.1%(36)	36.0%(18)	38.3%(18)
Educational status % (n)			
High School/GED or less	47.4% (46)	38.0% (19)	57.5% (27)
Annual Income % (n)			
< $20,000	68.0% (66)	70.0%(35)	65.9%(31)
Marital status % (n)			
Married or living with a partner	45.4%(44)	18.0% (9)	74.5%(35)
Employment status % (n)			
Full time or part time	32.0% (31)	32.0% (16)	31.9% (15)
Household Size *mean (sd)*			
Total children in the house	2.4 (1.3)	2.5 (1.4)	2.2 (1.1)
Total adults in the house	2.0 (1.0)	1.8 (1.2)	2.1 (0.8)
Food Assistance Use: *% yes (n)*			
SNAP	79.4%(77)	86.0%(43)	72.3%(34)
WIC	60.8%(59)	58.0%(29)	63.8%(30)
Food Security Status *% (n)*			
High or marginal	54.6%(53)	50.0%(25)	59.6%(28)
Low	28.9%(28)	30.0%(15)	27.7%(13)
Very Low	16.5%(16)	20.0%(10)	12.7%(6)
Grocery Shopping Trips *% (n)*			
Once a month	18.6% (18)	32.0% (16)	4.3% (2)
Twice a month	25.8% (25)	26.0% (13)	25.5% (12)
Three times a month	18.6% (18)	20.0% (10)	17.0% (8)
Four times a month or more	37.1% (36)	22.0% (11)	53.2% (25)
Family meals from fast food % (n)			
Never	34.0% (33)	34.7% (17)	34.0% (16)
1 time/week	33.0% (32)	32.0% (16)	34.0% (16)
2 times/week	24.7% (24)	30.6% (15)	19.1% (9)
3 times/week or more	8.3% (8)	4.1% (2)	12.7% (6)
Parent BMI			
BMI *mean (sd)*	31.3 (7.8)	31.4 (7.7)	31.2 (7.8)
BMI ≥30*% (n)*	47.4%(46)	50.0%(25)	44.7% (21)
Child BMI percentile % (n)			
< 5th percentile	5.2% (5)	10.0% (5)	0
5th - < 85th percentile	62.9% (61)	64.0%(32)	61.7% (29)
85th - <95th percentile	16.5% (16)	12.0% (6)	21.3% (10)
> = 95th percentile	15.5% (15)	14.0% (7)	17.0% (8)
Acculturation status (Hispanic/Latino only)			2.2(1.2)

*SCAN*   Study on Child Home Food Availability using TechNology, *BMI* Body mass index

There was an average of 2.4 children (SD: 1.3) and 2.0 adults (SD: 1.0) in each household. Most households reported earning less than $20,000/year and were receiving food assistance benefits (79.4% on SNAP, 60.8% on WIC). While the majority of households had high to marginal household food security status (54.6%), 28.9% and 16.5% households were in the low to very low categories of food security. Grocery shopping frequency differed somewhat by race/ethnicity. Thirty two percent of African American households shopped only once a month compared to 4.3% Hispanic/Latino households. Hispanic/Latino households were more likely to shop at least 3 times/month (70.2%) compared to African American households (42.0%). While 34% of households reported that they never purchased family meals from fast food restaurants, the remaining households reported purchasing family meals from fast food restaurants at least once a week. Among Hispanic/Latino households, the mean acculturation score was 2.2 (SD: 1.2) (Table 1).

### Anthropometrics

The mean BMI for parents was 31.3 kg/m^2^ (SD: 7.8). Almost half of the parents in this sample (47.4%) had a BMI of 30 or above. The majority of children in the study were between the 5th and 85th percentile for BMI (62.9%). However, a greater proportion of Hispanic/Latino children (38.3%) were at or above the 85th percentile for BMI compared to African American children (26.0%).

### Summary of diet quality indices

Table 2 reports means (SD) of total HEI-2010 scores and its subcomponents by race/ethnicity for parents’ diets, children’s diets, and the home food supply.

**Table 2:** Healthy Eating Index-2010 total and component scores of diets and home inventories by race/ethnicity

	Parent^a^				Child^b^				Home^c^			
	H		AA		H		AA		H		AA	
HEI-2010 Dietary Component (Max Score)	mean	SD	mean	SD	mean	SD	mean	SD	mean	SD	mean	SD
Total Fruit (5)	2.45	1.94	1.08	1.61	3.51	1.77	2.45	1.86	1.37	0.98	0.77	0.74
Whole Fruit (5)	2.62	2.15	0.88	1.76	3.09	1.88	1.85	1.98	1.94	1.26	1.11	1.05
Total vegetables (5)	3.06	1.62	2.31	1.32	1.64	1.18	1.58	1.11	1.84	1.05	2.21	1.03
Greens and beans (5)	2.01	2.20	1.13	1.82	0.89	1.73	0.67	1.41	0.92	1.19	1.26	1.50
Whole grains (10)	6.50	3.94	1.76	2.68	4.99	4.01	3.15	3.16	6.91	2.90	4.02	2.30
Dairy (10)	6.23	3.39	3.74	2.98	8.64	2.59	5.96	2.76	2.99	1.66	2.50	1.71
Total protein foods (5)	4.45	1.01	4.77	0.62	4.15	1.36	4.33	1.15	3.86	1.31	4.40	0.85
Seafood/plant proteins (5)	2.18	2.23	0.77	1.51	1.82	2.23	1.60	1.99	4.28	1.36	4.34	1.28
Fatty acids (10)	4.20	3.81	4.92	3.29	3.27	3.35	4.85	3.15	8.42	2.61	8.69	2.21
Refined Grains(10)	7.68	2.99	6.33	3.07	7.00	3.58	5.32	3.25	6.38	3.23	4.99	3.11
Sodium (10)	5.41	3.31	3.32	3.56	6.60	3.32	4.04	3.28	6.77	3.99	5.07	3.64
Empty Calories (20)	15.49	5.06	14.01	4.26	15.45	4.45	15.37	3.34	16.04	2.94	14.07	4.49
Total HEI Score (100)	62.27	13.93	45.01	11.03	61.05	13.89	51.17	12.61	61.73	11.41	53.42	9.57

Hispanic/Latino (H) *n* = 47 parent-child dyads; African American (AA) *n* = 50 parent-child dyads. HEI scores for dietary intake are based on the average of diet recalls from home visits 1 and 2. ^a^Number of parent diet recalls collected at home visit 1: 97; home visit 2: 95. ^b^Number of child diet recalls collected at home visit 1: 97; home visit 2: 94. HEI scores for home food inventories are based on the average of home inventories from home visits 1 and 2. ^c^Number of home food inventories collected at home visit 1: 97; home visit 2: 94. In households with missing data, available data from home visit one was used in estimates instead of averaging data

#### Parent diet quality

**Fig. 2:**
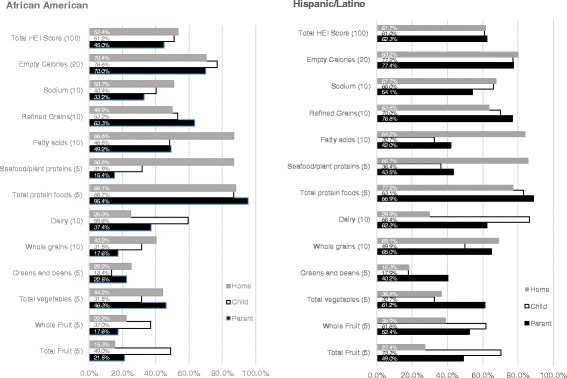
Percentage of maximum score of Healthy Eating Index-2010 components (total and subcomponents) for diets of parent-child dyads and home food inventories by race/ethnicity

The total HEI-2010_indivdiual-level_ scores were higher among Hispanic/Latino parents (mean: 62.27 SD: 13.93) than African American parents (mean: 45.01 SD: 11.03) in this sample. The only category where both groups nearly reached the maximum score was for *total protein foods* (Hispanic/Latino: mean 4.45 SD: 1.01; African American: mean: 4.77 SD: 0.62) (Table 2). Among African American parents, the three HEI subcomponent scores that were furthest away from meeting the maximum score (% of maximum score) were: *seafood and plant proteins* (15.4%), *whole grains* (17.6%), and *whole fruits* (17.6%) (Fig. 2). Among Hispanic/Latino parents, the three HEI subcomponent furthest from maximum scores were *greens and beans* (40.2%), *fatty acids* (42.0%), and *seafood and plant proteins* (43.5%) (Fig. 2).

#### Child diet quality

Children’s diets followed a similar pattern in that Hispanic/Latino children had a higher mean total HEI-2010_indivdiual-level_ score than African American children in this study (Hispanic/Latino: mean: 61.05 SD: 13.89; African American: mean 51.17 SD: 12.61) (Table 2). Among African American children, the three HEI subcomponent scores that were furthest away from meeting the maximum score were *greens and beans* (13.4%), *whole grains* (31.5%), and *total vegetables* (31.5%) (Fig. 2). The HEI-2010_indivdiual-level_ subcomponent scores furthest from achieving the maximum for Hispanic/Latino children were *greens and beans* (17.9%), *fatty acids* (32.7%), and *total vegetables* (32.7%) (Fig. 2).

#### Diet quality of home food supply

Consistent with individual level diet quality, the total HEI-2010_home-level_ score was higher among Hispanic/Latino households (mean 61.73 SD: 11.41) compared to African American households (mean 53.42 SD: 9.57). Among African American households, the three HEI-2010_home-level_ sub component scores furthest from the maximum score were *total fruit* (15.3%), *whole fruit* (22.2%), and *greens and beans* (25.2%) (Fig. 2). For Hispanic/Latino households, the lowest HEI-2010_home-level_ were also for *greens and beans* (18.3%), *total fruit* (27.4%), and *dairy* (29.9%) (Fig. 2).

### Relationship between individual level diet quality and diet quality of the home food supply

Table 3 reports the results of models examining associations between individual level diet quality and home-level diet quality. The unadjusted model examining parent diet quality (dependent variable) with home-level diet quality (independent variable) produced a beta coefficient of 0.58 (95% CI: 034–0.83). After controlling for potential confounding factors, the adjusted estimate was 0.36 (95% CI: 012–0.60) (Table 3). This suggests that a one-point increase in home-level diet quality corresponds to a 0.36 point increase in parent diet quality. The adjusted model that examined children’s diet quality with total diet quality at the home level produced comparable estimates (0.38 95% CI: 013–0.62). As for subcomponent scores, positive associations were observed between individual-level and home level diet quality for *total fruit* (parent: 0.55 95% CI: 0.16–0.94; child: 0.49 95% CI: 0.03, 0.94), *whole fruit* (parent only: 0.41 95% CI: 0.07–0.74), *greens and beans* (parent only: 0.39 95% CI: 0.05–0.74), and *whole grains* (child only: 0.33 95% CI: 0.04–0.63).

**Table 3:** Associations between individual level diet quality and the diet quality of the home food supply

	Parent (*n* = 97)				Child (*n* = 97)			
	*unadjusted*		*adjusted*		*unadjusted*		*adjusted*	
	β	*95% CI*	β	*95% CI*	β	*95% CI*	β	*95% CI*
Total Fruit	0.77	(0.38,1.16)^a^	0.55	(0.16, 0.94)^b^	0.70	(0.31, 1.09)^a^	0.49	(0.03, 0.94)^c^
Whole Fruit	0.56	(0.22, 0.89)^b^	0.41	(0.07, 0.74)^c^	0.34	(0.01, 0.66)^c^	0.20	(−0.14, 0.54)
Total Vegetables	0.08	(−0.21,0.37)	0.24	(−0.10, 0.59)	0.03	(−0.19, 0.24)	0.01	(−0.24, 0.27)
Greens and Beans	0.28	(−0.02, 0.58)	0.39	(0.05, 0.74)^c^	0.07	(−0.16, 0.30)	0.01	(−0.25, 0.26)
Whole Grains	0.56	(0.31, 0.82)^a^	0.22	(−0.04, 0.49)	0.42	(0.18, 0.65)^b^	0.33	(0.04, 0.63)^c^
Dairy	0.18	(−0.21, 0.59)	0.02	(−0.48, 0.53)	0.01	(−0.35, 0.36)	0.07	(−0.34,0.49)
Total Protein	0.03	(−0.12, 0.18)	−0.03	(−0.22, 0.16)	0.16	(−0.06, −0.38)	0.21	(−0.05, 0.48)
Seafood and Plant Protein	0.12	(−0.19, 0.43)	0.15	(−0.17, 0.48)	0.26	(−0.53, 0.58)	0.20	(−0.15, 0.55)
Fatty Acids	−0.10	(−0.40, 0.20)	−0.18	(−0.52, 0.16)	0.22	(−0.06, 0.49)	0.26	(−0.05, 0.57)
Refined Grains	0.06	(−0.14, 0.25)	0.09	(−0.12, 0.29)	0.22	(0.003, 0.43)^c^	0.20	(−0.04, 0.43)
Sodium	0.15	(−0.04, 0.33)	0.11	(−0.11, 0.29)	0.17	(−0.01, 0.35)	0.08	(−0.10, 0.27)
Empty Calories	0.25	(0.02, 0.48)^c^	0.23	(−0.02, 0.49)	0.02	(−0.18, 0.22)	0.02	(−0.19, 0.23)
Total Diet Quality	0.58	(0.34, 0.83)^a^	0.36	(0.12, 0.60)^b^	0.49	(0.26, 0.72)^b^	0.38	(0.13, 0.62)^b^

Healthy Eating Index 2010 (HEI-2010) was used to assess diet quality at the individual- and home- level. Dependent variable: Individual level HEI-2010 total and subcomponent scores, respectively. Independent variable: Home level HEI-2010 total and subcomponent scores, respectively. Adjusted models controlled for race/ethnicity, age (parent and child), education, marital status, employment status, income, food assistance, body mass index (parent and child), household food security, grocery shopping frequency, family food meals frequency. ^a^ *p* <0.001, ^b^*p* <0.01, ^c^ *p* <0.05

## Discussion

A primary objective of the SCAN study was to examine the relationship between individual-level total diet quality and home-level total diet quality as assessed through direct observation of the home food supply in the households of low-income African American and Hispanic/Latino families with preschool-age children. Overall, this study found that individual-level total diet quality was positively associated with home-level total diet quality for both parents and children, respectively.

This study used the HEI-2010 to assess the quality of diets of parent-child dyads. Total HEI-2010_indivdiual-level_ scores (parent mean: 53.37; child mean: 55.96) are comparable with estimates from NHANES data (58.27 for adults 18–64; 55.07 for children 2–17 years) [13]. This current study also found diet quality scores to be higher among Hispanic/Latino households compared to African American households, which is consistent with nationally representative data. For example, Gu et al. examined NHANES data from 2011 to 2012 and found HEI-2010 total diet quality scores to be highest among Mexican Americans and lowest among Non-Hispanic Black participants for both adults and children [14]. Given that Hispanic/Latino and African American households in this present sample were similar across socioeconomic indicators (e.g. income, employment status); differences in diet quality are more likely attributable to culture and food preferences rather than other factors. Acculturation has been noted as a determinant of diet quality among certain Hispanic/Latino subgroups [64–66]. Based on NHANES data (1999–2012), Mexican Americans with lower acculturation scores had better odds of higher overall diet quality status (HEI-2010) and in subcomponents of diet quality(i.e. fruits, vegetables, sodium, and empty calories) [67]. This provides support for our findings in that our study sample is largely of Mexican descent and less acculturated (Table 1).

Other studies have conducted home food inventories by scanning barcodes [35–37], but ours was the first to apply the HEI to examine diet quality at the individual- and home-level. For instance, Byrd-Bredbenner and colleagues examined nutrient availability in households using the nutrient adequacy ratio (NAR) [68, 69]. In contrast to the HEI-2010, the NAR measures nutrients to be maximized (e.g. vitamins A, C, protein, fiber, iron, and calcium) and minimized (e.g. total fat, cholesterol, sodium, and total sugar), but does not directly measure food groups. They found differences by SES in that higher SES non-Hispanic White (NHW) households were closer to meeting maximum scores for nutrients to be maximized compared to lower SES African American and Mexican (from Oaxaca) households. Among the lower SES households, African American homes had lower nutrient availability scores than Mexican households [40]. Similarly, home-level diet quality scores were also lower in African American households in our sample compared to Hispanic/Latino households. However, household-level nutrient availability was not examined in relation to dietary intake in the earlier study [40], which limits the ability to make further comparisons.

Stevens and colleagues developed the “Exhaustive Home Food Inventory” method which used handheld UPC scanners to inventory nearly all food items in the homes of 80 low-income African American first-time mothers with infants [36]. While they did not examine diet quality using an index (e.g. HEI, NAR, etc.), they did assess the home food supply relative to fruit and vegetable consumption. They found a positive association between home availability of fruits and vegetables and fruit and vegetable intake among infants, but not mothers. [38]. We also observed a positive relationship between home availability of fruits and fruit intake, but our findings applied to both parents and children. In contrast, our sample included Hispanic/Latino households and slightly older children. In addition, we did not see a relationship between home availability of total vegetables and vegetable intake, but parent’s intake of greens and beans was associated with home availability of those foods.

In another approach for assessing the home food environment, Appelhans et al. applied the HEI-2010 to examine the quality of foods in the home by collecting household food receipts (e.g. grocery stores, fast food/carryout, restaurants, other) [62]. They reported a median total HEI-2010 score of 60.9, which is comparable to the median score of our Hispanic/Latino households (60.8), but higher than the African American households (median: 54.0) (data not shown) in our sample. They found a positive correlation between total HEI-2010 scores based on household receipts and total HEI-2010 based on 24-h diet recalls. This finding is consistent with ours despite differences in home food availability assessment and sample demographics. They also found stronger associations for fruits, vegetables, and fiber. Findings from our study are comparable in that stronger associations were observed for total fruit (parent and child), greens and beans (parents only), and whole grains (children only). It is interesting that a similar observation was not found with moderation components such as empty calories, sodium, and refined grains. Further studies are needed to confirm this result (e.g. larger study sample). Additional research is also needed to determine how diet quality, measured at the level of the environment (e.g. home) relates to health outcomes or other health-related behaviors.

A primary strength of this study is that we used an objective and comprehensive method to assess the home food environment. This method makes no prior assumptions about a household’s current environment because it measures what is actually in the home rather than what we think should be available based on a predefined list (e.g. checklist). This method is well suited for learning about contexts that are not well understood. Furthermore, this approach makes it possible to apply a diet quality index such as the HEI-2010; thus making it easier to examine how diet quality at the home level relates to dietary intake of household members. Despite these strengths, there are limitations to consider. Due to the cross sectional nature of this study, it is not possible to infer causality between diet quality at the home level to diet quality at the individual level. While technology has made it easier to conduct nearly exhaustive home food inventories, this methodology can still be both labor and time intensive. Also, in its current state, this method can only be reliably carried out by trained research staff and not study participants, which may limit the wide scale use of this approach. Although multiple inventories and recalls were collected to capture intra-individual variation, some food items might have been consumed before they could be inventoried. Lastly, only low-income African American and Hispanic/Latino households with young children living in an urban setting were enrolled in this study; therefore, generalizability of these findings to other populations may be limited.

## Conclusion

One way to address diet-related disparities is to impact the home food environment. Efforts to influence this environment requires a clearer understanding of the home setting. This study found that overall diet quality at the individual was positively related home level diet quality. These findings offer an objective and comprehensive approach for assessing the diet quality of the home food environment in low SES minority households. The home environment can provide useful formative information to guide intervention approaches targeting populations at greater risk for poor dietary intake. A next step for this research would be to test the effectiveness of a home-based dietary intervention that uses home-level diet quality scores assessed at baseline to inform intervention development.

## Abbreviations

BMI: Body Mass Index
CSV: comma-separated values
DGA: Dietary Guidelines for Americans
HEI-2010: Healthy Eating Index-2010
NCC: Nutrition Coordinating Center
NDSR: Nutrition Data System for Research
NHANES: National Nutrition and Health Examination Survey
SNAP: Supplemental Nutrition Assistance Program
UPC: Universal Product Codes
USDA: United States Department of Agriculture
WIC: Special Supplemental Nutrition Program for Women, Infants, and Children
